# What factors influence successful recruitment of siblings of individuals with first episode psychosis to e‐health interventions? A qualitative study

**DOI:** 10.1111/hex.12508

**Published:** 2016-10-06

**Authors:** Jacqueline Sin, Claire Henderson, Debbie Spain, Catherine Gamble, Ian Norman

**Affiliations:** ^1^ Health Service & Population Research Department Institute of Psychiatry, Psychology & Neuroscience King's College London London UK; ^2^ MRC Social, Genetic and Developmental Psychiatry Centre Institute of Psychiatry, Psychology & Neuroscience King's College London London UK; ^3^ South West London & St George's Mental Health NHS Trust London UK; ^4^ Florence Nightingale Faculty of Nursing & Midwifery King's College London London UK

**Keywords:** brothers and sisters/siblings, e‐health interventions, families/family carers, psychosis, trial recruitment

## Abstract

**Background:**

Recruitment to clinical research studies can prove complex. This is particularly true of mental health research, given factors such as confidentiality, capacity and consent, or when attempting to recruit family members as opposed to service users themselves.

**Aim:**

This study investigated the challenges experienced and strategies employed in the recruitment of siblings of people with first episode psychosis using Early Intervention in Psychosis Services (EIPS) in England.

**Methods:**

As part of a randomized controlled trial (RCT) of an e‐health intervention for siblings, we conducted a process evaluation study whereby semistructured interview was undertaken with clinical and research staff involved in recruitment of siblings. Data were analysed thematically.

**Results:**

Twelve participants from six EIPS were interviewed. Data analysis revealed seven key themes: (i) limited comprehensive family data available; (ii) data governance and consent issues; (iii) organizational factors; (iv) convoluted recruitment methods; (v) concerns about service users' opinions; (vi) fluidity in siblings' needs and expectations; and (vii) strategies to enhance recruitment.

**Conclusions:**

Recruitment challenges identified in this study concerned administrative, organizational, process and attitudinal issues. These are similar to other studies recruiting mental health service users as well as family members. Failure to recruit to target implies that studies are underpowered to detect potential statistically or clinically meaningful changes. Future studies should establish how best to enhance family inclusiveness in clinical practice and research.

## 1 Introduction

Siblings of individuals with first episode psychosis (FEP) are vulnerable to developing mental health problems, partly due to the impact of psychosis on individuals within the family, and the wider network.^1^, ^2^, ^3^ However, siblings also have an important role in supporting service users' recovery and enhancing prognosis.^4^, ^5^ Recent evidence has indicated that siblings can benefit from information about psychosis, as well as strategies to enhance coping, and opportunities for peer support.^3^, ^5^, ^6^, ^7^ Hence, the E Sibling Project was developed to provide a FEP sibling‐specific intervention, using an Internet‐based medium for flexible access and individualized package.^6^

The study described here formed part of the process evaluation for the E Sibling Project Randomised Control Trial (RCT) (See also Sin et al.,^6^, ^7^ for information regarding development and usability establishment of the intervention). The E Sibling Project RCT recruited siblings (either biologically related, step‐ or half‐siblings or related through adoption) of individuals who were using Early Intervention in Psychosis Services (EIPS) in England, between September 2013 and March 2015. EIPS typically provides comprehensive care using an assertive outreach approach for people aged between 18 and 35, experiencing FEP.^8^ EIPS commonly promotes family inclusiveness, such as through providing family intervention, and enhanced psychoeducation for family carers.^8^, ^9^

The RCT aimed to recruit 144 siblings, in 9 months (September 2013 to April 2014) originally; the sample size was estimated in order to ensure there was sufficient statistical power to detect treatment effect. At the time of undertaking this process evaluation study (April to July 2014), siblings were recruited from 16 EIPS across England, with a combined caseload of approximately 5000 service users. We were mindful that there could be challenges in recruiting to target, due to uncertainty experienced by family members in engaging with mental health services,^10^, ^11^ stigma about being involved,^12^ or competing demands.^13^, ^14^, ^15^, ^16^ Hence, we had set a conservative recruitment rate based on previous regional surveys,^1^, ^2^ suggesting that 90% of EIPS service users would have one sibling or more. We hypothesized that at least 50% of siblings would meet the RCT eligibility criteria (living in England, aged 16 or above, and in weekly or regular contacts with service users), based on consultation with Principal Investigators (PIs) and EIPS leads in five recruitment sites as part of our initial trial setting up preparation.^6^ Our sampling frame comprised 2250 siblings. Our recruitment procedures involved (i) potentially eligible siblings being identified by local clinicians or research staff in each participating EIPS, (ii) siblings and/or service users and their named carers being given study information from the clinicians or researchers, (iii) then siblings could self‐refer or be referred through the local personnel to join the E Sibling Project. Study information materials (localized for each participating EIPS) were devised for direct communication with siblings if they were already known to the service, as well as for informing service users or named carers who were often parents, in order to ask them to help pass the study information to siblings. These recruitment procedures were devised to ensure that service users were informed about the study as is good practice even though they were not required to participate in the study nor to give consent for their siblings to join the study (of note, ethical approvals were in place for such).^6^, ^7^

By April 2014, 9 months after the RCT commenced and at the end of our original recruitment period, 58 siblings (40% of the total required) had been recruited. We instigated some strategies as contingency plan to overcome the recruitment shortfall. These included: extending the recruitment period to March 2015; increasing the number of recruitment sites; and undertaking this process evaluation study with clinical and research staff involved in the recruitment of siblings. Understanding factors associated with recruitment, including those that facilitate as well as impede recruitment, is important in order that intervention trials are feasible. To explore the recruitment strategies, and factors that potentially mediated successful recruitment, this study investigated “reach” and “recruitment,” using the components of process evaluation as outlined by Steckler & Linnan.^17^ Reach was defined as the extent to which participants were made aware of RCT recruitment, either directly or indirectly (eg via other family members or service users);^17^ investigation of recruitment included examining intended and actual recruitment procedures, as well as practical difficulties experienced in recruiting participants.

## 2 Methods

### 2.1 Study design

Qualitative semistructured interviews were undertaken with clinical and research staff involved in recruiting siblings into the RCT. Also, we calculated the number of service users per EIPS caseload, the proportion of service users known to have siblings, and the number of siblings offered information about the study. These figures were compared with the number of individuals who consented to take part, so as to estimate reach of the recruitment activities and service recruitment rates.

### 2.2 Regulatory approvals

Approvals were granted by the National Health Service (NHS) Research Ethics Committee (Reference: NRES 12/LO1537), and by local Research and Development (R&D) departments at participating NHS Trusts. All participants provided written informed consent.

### 2.3 Participants

We recruited clinical and research staff, including local PIs who were NHS senior clinicians or clinical academics involved in leading local recruitment; or Research Assistants (CSO/CSA) funded by the Clinical Research Network: Mental Health (CRN: MH) to support recruitment from mental health services,^18^, ^19^ and clinicians who worked at EIPS. As the first step to identify the potential participants, we informed the PIs in all the recruitment sites about the process evaluation study. Second, we asked for a list of clinical and research staff who were involved in recruiting siblings locally. Lastly, we circulated the study flyer and participant information sheet to all the potentially eligible participants.

### 2.4 Data collection

A topic guide (available from the first author) was developed and piloted, in consultation with the E Sibling Project Sibling Reference Group (The SRG comprised five siblings to provide oversight for the project design and conduct)^20^ and the West Midlands Carer Reference Group (The CRG is a Department of Health‐funded carer group which meets monthly to give comments on studies focusing on mental health informal carers in England).^21^ Questions were designed to prompt participants about their experiences, successful or otherwise, in identifying, approaching and recruiting siblings. Interviews were conducted in person or by phone, depending on participant's preference.

### 2.5 Data analysis

Interviews were transcribed and analysed using Ritchie et al.'s^22^ thematic analysis framework, in Nvivo version 10 (http://www.qsrinternational.com). The analysis process comprised three stages. First, initial themes were identified by “indexing” the transcript; these themes guided the formation of a framework within which transcribed material was summarized. Second, key categories were identified to describe the data. Finally, patterns of association were sought. Data analysis was performed in conjunction with data collection, in order that the developing framework could be compared, contrasted and validated until data saturation was reached.^23^

## 3 Results

### 3.1 Participants' professional characteristics and recruitment experience

Four PIs, four CSOs/CSAs and four clinicians (total n=12), working in six EIPS, were interviewed (See Table 1). Interviews were audio‐recorded, and conducted by phone (n=6) or in person (n=6). Interview duration ranged between 21 and 70 minutes (average duration=40 minutes; median=34.5 minutes).

**Table 1:** Summary of participants' professional characteristics

	PIs	CSOs/CSAs	Clinicians
Length of time in current positions	2‐10 years	5 months‐3 years	4‐9 years
Professional background/positions	EIPS manager ‐ 1Consultant ‐ 1FI coordinator ‐ 1Research manager ‐ 1	CSO ‐ 2CSA ‐ 2	Occupational Therapist ‐ 1Mental Health Nurses ‐ 2Clinical Psychologist ‐ 1
Age range	45‐54	24‐54	30‐38

FI coordinator, family intervention coordinator; CSO, clinical studies officer; CSA, clinical studies assistant; EIPS, early intervention in psychosis services.

With the exception of three participants, there was consensus that clinical research regularly took place in EIPS, with staff recruiting to approximately 10 to 15 studies during the past three years. Studies predominantly included EIPS service users or staff; family members, including siblings, were seldom recruited. While family‐focused research took place occasionally, participants reported that the emphasis was on recruiting service users. Conversely, family members were approached primarily to provide informant ratings of service users' symptoms, and occasionally about their own health outcomes.

### 3.2 Themes

Seven key themes were identified in the analysis, which concerned the process and challenges encountered with respect to recruitment of siblings of service users with FEP into the E Sibling Project RCT. These were as follows: (i) limited comprehensive family data available; (ii) data governance and consent issues; (iii) organizational factors; (iv) convoluted recruitment methods; (v) concerns about service users' opinions; (vi) fluidity of siblings' needs and expectations; and (vii) strategies to enhance recruitment. See Table 2.

**Table 2:** Summary of challenges and strategies to enhance recruitment

Themes of recruitment challenges	Examples of challenges	Proposed strategies to promote recruitment
Limited comprehensive family database available	ECR carry incomplete family detail ECR focuses on one named carer only	Comprehensive assessment on families and carers Constructing genogram as standard Review ECR and recording method
Convoluted recruitment methods	Indirect approach to siblings Limited access to siblings through service users	Provision of study information in various formats and media, eg written, electronic Direct approach to siblings through primary care and voluntary sectors
Concerns about service users' opinion	Presumed lack of interest in clinical research Illness and symptoms prohibit information‐giving on research	Staff training PPI in study design and information leaflets Promotion of good practice guidance in recruiting mental health service users and carers into research
Fluidity in siblings' needs and expectation	Siblings (& families in general) perceived as difficult to engage in research Competing demands in siblings' life	Taking on board siblings' (families in general) needs in optimizing research design Explaining research aims clearly Arranging reminders of invitation to study
Organizational factors; data governance and consent issues	Working alliance between clinicians and CSOs/CSAs Lack of recognition and reward for frontline staff	Guidelines on using carer database to inform them about clinical research Identifying a research champion within each team Reinforcing working relationship and communication between clinicians, CSOs/CSAs, R&D personnel Support a positive clinical research culture that integrate clinical and research agenda and activities

ECR, electronic case record; PPI, patient and public involvement; CSO, clinical studies officer; CSA, clinical studies assistant; R&D, research and development.

#### 3.2.1 Limited comprehensive family data available

There was unanimous consensus between participants that in each service, there were limited data about the family structure and relationships between service users and their immediate family members (ie the degree to which service users were in contact with family members). Furthermore, even when named carers were identified, more often than not, their contact details were not recorded or incomplete. Additionally it was noted that most carers were parents. Hence, even when CSOs/CSAs were able to access electronic records to screen for potentially suitable individuals, there were no straightforward ways to identify siblings. This difficulty is illustrated by one participant:

I feel like I'm forever chasing up the information (from clinicians) that just doesn't get back to you so I would try to do what I can through the information on RiO (a common electronic record system used in NHS) but again that's very limited a lot of the time so it's not been easy in that aspect.

(Trust 3, CSA C)

#### 3.2.2 Data governance and consent issues

Several participants, including EIPS clinicians and CSOs/CSAs, described ambiguity in Trust data governance policies regarding whether it was possible to send siblings information about the study. While these details were expected to be obtained as part of a standard needs assessment, it seemed unclear whether named individuals had in fact consented to be contacted for any information about research or services. One manager suggested that the clinicians would need to contact each carer individually to seek their consent for any study information to be sent to them. Meanwhile, contacting carers to clarify whether they wanted any information about research was understandably not considered to be the top priority for clinicians. A participant interviewed empathized with these concerns:

It's the interpretation of the increased focus on information governance, … I think the push on information governance has really diluted the impact that clinicians have on liaison with anyone apart from the service users themselves so it's potentially seen as a bit of a barrier and I think clinicians have automatically felt that it's made it more difficult to have direct contact with a family member without direct consent from the service user themselves.

(Trust 2, PI G)

Confusion and uncertainty regarding the data governance framework seemed to further demotivate some clinicians and CSO/CSAs to explore ways to approach the families/siblings directly. Nevertheless, there were two cases reported by the participants themselves that they gave the study information directly to the siblings. One of the siblings was the named carer for her unwell brother; the other sibling lived with her brother and parents but felt she was in need of more information and support for herself. In both scenarios, the senior clinicians (also PIs in the respective sites) deemed it appropriate to inform the siblings about the study directly.

In addition, several clinicians expressed concerns about whether approaching siblings directly, without obtaining consent from the service user first, would breach data governance and confidentiality policies. The accounts given by participants suggested that clinicians would often err on the side of caution, as illustrated by the following quotation:

I believe we should go through the service users first as ultimately they are our clients, not their families. It's difficult in a couple of cases as I do know their siblings would be suitable for it and would grab the opportunity to try the resource with both hands, but I can't really talk to them without letting the service users know and I know the service users won't like it.

(Trust 5, Clinician F)

#### 3.2.3 Organizational factors

A number of organizational and infrastructural factors were identified as impeding the integration of clinical and research agendas. The most commonly cited reason that dissuaded clinicians from being more enthusiastic in study promotion activities was the sheer volume of their clinical and administrative workloads. One participant described this dilemma:

They (clinicians) are too busy, they're barely getting their notes, risk …The demand has increased and the resources haven't alongside it and that means that then have to go to another meeting about something else, like research, going and having to talk to the client about a study we just haven't got time to do it.

(Trust 1, PI H)

Frontline staff appeared to consider research‐related work as an addition to their workload, or outside of their remit. This was often compounded by a commonly held perception of a lack of support, recognition or reward, from the Trust or senior management. These views are illustrated by one participant:

A lot of things need to change from the top—such as giving clinicians time and opportunities to get involved in research, be able to tap into some of the research income for staff to buy books or resources, do training or go to conferences—at least to know their efforts have been recognised and rewarded.

(Trust 2, PI G)

Support from Trust‐employed CSOs/CSAs was available in all participating Trusts for recruitment activities, but the working relationship between clinicians and CSOs/CSAs varied widely. More often than not, CSOs/CSAs were not seen as part of the clinical service, and hence, clinicians did not feel comfortable for them to contact service users (or their families). One clinician described her reasons for not accepting assistance from the CSA:

Even though we have the gentleman (the Trust‐employed CSA) helping with mailshot or talking to people, I don't think that would work, I think it's more about it's you (who are the clinician/care coordinator) have got that therapeutic relationship and an understanding with the service users and the family.

(Trust 1, Clinician E)

#### 3.2.4 Convoluted recruitment method and unintended selective sampling

We contacted the local research and clinical staff on a monthly basis to check the number of potentially suitable siblings and/or service users (or the named carers) who had been identified and any of them were given information about the study. We also offered to follow up on any potentially interested participants to answer any queries they might have, if they had agreed for the researchers to contact them. It was reported that approximately 10% of each clinician's caseload comprised service users who had siblings eligible for the study, contrary to our estimation of at least 50%. In an average caseload of 15 service users (per care coordinator) among EIPS clinicians, it was common that clinicians identified only one or two service users who they thought might have siblings eligible for the study. The most common recruitment route involved EIPS clinicians mentioning the study to service users, and asking them to pass on information (NHS research ethics approvals permitted both direct approach to siblings and indirect approach to siblings via service users or the named carers). In most services, this was the sole recruitment method used, as described by one participant:

Even we did extra things to get research ethics to approach everyone (family members and siblings) as direct as possible, but at the end actually it didn't change clinicians' perception that the only route they can see is talking to the service users.

(Trust 1, CSA D)

This might have resulted in additional sampling filters based on clinicians' judgement of service users' factors and other issues. Clinicians identified potentially suitable service users (and siblings) predominantly based on their knowledge of, or contact with service users' siblings. This seemed to result in selective identification of potentially eligible siblings with whom the clinicians had had direct contact with (eg seen at care planning meeting) or those siblings who lived together with the service users. That is, clinicians would naturally have more contacts with these siblings and somehow would find it easier to give them the study information directly or indirectly through the service users. As a consequence, siblings who were not living under the same roof with their unwell brother/sister but also provided a lot of support in their care outside of the office hours (such as visiting over weekends or evenings) would be less likely to be informed about the study. Some reasons behind such an unintended selective sampling process were exemplified by one participant:

I think that it can be quite hard for clinicians for lots of reasons because often siblings are still at school or work or at university in another town when they are visiting, there're all the different things and … I think that might be people don't take it to that level really to make that personal contact.

(Trust 4, PI J)

#### 3.2.5 Concerns about service users' opinions and responses to study information

Participants raised understandable concerns about how service users might think and feel about their siblings being recruited to a study in which they were not involved. Also, it was noted that some service users were viewed as vulnerable, or floridly symptomatic (eg paranoid or delusional), which meant that clinicians did not wish to mention information that could be misconstrued or potentially exacerbate suspiciousness. Several participants mentioned that service users could seem sensitive to changes in their routines, for example, meeting unfamiliar researchers, including the CSOs/CSAs. Consequently, it transpired that clinicians could tend to avoid conveying information about the study out of the intention of not adding unnecessary pressure onto the service users. One CSO described his observation:

Well they (the clinicians) make a judgement on their (service users) behalf. It's the same with, not just yours but with other studies, as soon as they start discussing their patients there are “ifs” and “buts”, oh I don't know about this because at the moment I don't think they're settled; they've just moved to a new house. They seem trivial little things that seem to get in the way as to why they wouldn't pass the name over (for further information to be sent).

(Trust 1, CSA D)

While the number of service users identified by clinicians or CSOs/CSAs as potentially suitable for obtaining further study information was small, it was suggested that there was further screening out process prior to information to be shared. Some CSOs/CSAs interviewed seemed to hold negative beliefs about mental health service users' interests and capabilities in involving in research (in the case of the E Sibling Project, to pass the study information to their siblings) due to their illness and symptoms. These concerns are illustrated by one participant:

Especially obviously if there are things like psychosis at hand I wouldn't (talk to the service users) because it just seems as though it's something that they wouldn't be interested in, of concern to them, etc. I wouldn't say they necessarily would be aware of what was going on with their sibling or with their carer because their world is very limited.

(Trust 2, CSO F)

#### 3.2.6 Fluidity in siblings' needs and expectations

In general, study participants perceived family members of individuals with FEP to have needs that were distinct from those of individuals who have longer term difficulties. For example, family members typically had had no or minimal contact with mental health services, and as such were less familiar with services available, and unclear about how best to advocate for their needs. It was reported that issues associated with diagnostic ambiguity and prognostic fluidity could affect recruitment to research as well as engagement with the service. Participants identified that many of the EIPS carers (and siblings) might also be preoccupied with dealing with crises which arose with the FEP, therefore would often prioritize their attention on the service users rather than themselves. Some might be adopting a wait‐and‐see or a so‐called sealing over approach hoping that it was an one‐off episode and that they would not need further input from the mental health service beyond the current episode. The paradox was highlighted by one EIPS clinician:

I think they (EIPS families) are more difficult in the sense of the time factor—that's not relevant to us just now, or everything is going to be fine now, some may think it's just a one off and they don't necessarily see the vulnerability side of it.

(Trust 1, Clinician E)

Overall, parents were described as seeming positive about the study when they were asked to pass on information to their other children. Yet, several clinicians stated that a minority of parents appeared suspicious and less amenable to being involved in recruitment. Clinicians believed that this might be because parents might be experiencing a sense of denial about their child's illness, or they might be trying to shield their other children from having any contact with the mental health service. In comparison, while some siblings were identified by clinicians as being potential study participants, and keen to take part when first given the information, it transpired that a significant proportion did not pursue this further, which was reported to be in part attributed to the demands of everyday life. One participant offered her observation:

I can see because that's just like normal things in life isn't it? or if you are working and doing some study it's like trying to stretch your time and motivate yourself to do things and to start that essay or to do whatever it is that you need to do.

(Trust 3, Clinician D)

#### 3.2.7 Strategies to enhance recruitment

All participants mentioned strategies to enhance integration of research activities into routine clinical practice. Infrastructural factors such as enhancement of cohesive working relationships between research and clinical staff and, potentially, direct remuneration to teams involved in research activities, were suggested. Having a clinician identified as a research champion within each team was also deemed good practice, whereby they could link with the PI as well as CSOs/CSAs, and R&D. One clinician elaborated on this:

I think a certain amount of dialogue with the team … which is obviously the role that I took and keeping it in people's minds fresh, … It probably helped because I am a care coordinator and yet I was advocating saying come on guys this is really important and just reminding people. And I communicate closely with the CSA and the PI on behalf of the team.

(Trust 3, Clinician D)

Training and supervision were also identified as having the potential for changing attitudes of the workforce. Few staff had experience of recruiting family members; fewer still had received training in this. None of the participants were aware of any particular good practice guidelines in involving family members (in their own right) in clinical research. Many participants suggested that there was a long way to go before families and carers were fully incorporated into mental health research programmes in a similar way that they would be currently incorporated into research about general health, older adults or children's services. One PI commented:

I think the best bit about the E Sibling Project is it's unearthed all these but I think it's only the tip of the iceberg, I think there is a whole layer of systems and attitudes and beliefs about who actually helps people's recovery. I don't think in anything I've read really articulates that well.

(Trust 3, PI B)

In two recruitment sites, family intervention (FI) was integral to the EIPS and training on working with families and carers was widely available. Suggestions were made to incorporate considerations for supporting siblings and promoting research activities in such training targeting clinicians and support workers. This suggestion was elaborated by a participant:

Since this year, whenever we run the FI training, we have a session focusing on siblings—raise awareness of their needs and their significance, and most importantly introduce the novel ideas including the study…. Clinicians instantly see the value of it, and that encourages clinicians to reach out for siblings who may not be able to come to the family work sessions as they live away.

(Trust 6, Clinician L)

## 4 Discussion

Given the multilayered recruitment process, and various obstacles encountered, it was not possible to ascertain the extent of reach of these recruitment activities to siblings. During a 12‐month period (September 2013 to August 2014), only 40% of the target sample of siblings (n=58) were recruited from a total caseload of 5000 service users covered by the 16 EIPS. Nonetheless, in reality, it seems likely that only a fraction of siblings of these service users were informed of the study. Many obstacles and challenges were described in terms of the identification and recruitment of siblings to the E Sibling Project RCT. These recruitment figures are comparable to studies which have recruited family members from similar^3^, ^5^, ^10^ and broader mental health settings.^13^, ^24^, ^25^

Several of the challenges encountered were, in some terms, anticipated and similar to those experienced by most clinical trials. For instance, despite the NHS pledge to prioritize clinical research,^26^, ^27^ an array of organizational and infrastructural barriers have repeatedly been identified as hampering progress towards this objective. These include the following: lack of integration of research agendas and activities within clinical settings; breakdown in communication between R&D, governance bodies and clinicians; lack of redistribution of gains, returns or research income to frontline services; lack of dedicated time for clinicians to get involved in research, compounded by the pressure from increased workload; and frequent delays in starting recruitment in clinical areas.^14^, ^15^, ^28^, ^29^, ^30^, ^31^, ^32^, ^33^

Study participants also outlined some mental health specific challenges. A general impression was conveyed that mental health clinicians can seem protective of their caseload and may hold pessimistic assumptions about service users' capacity and motivation to become involved in research activities, a finding which has been reported elsewhere.^14^, ^15^, ^28^, ^30^ For example, one recent study investigated recruitment rates of mental health service users to trials found that only 17% (n=131/752) of potentially eligible service users were approached by clinicians.^16^ This potentially implies that clinicians may feel reticent to approach service users, thereby indirectly influencing recruitment to research, before service users are even made aware of the possibilities of involvement in research activities.^16^ Despite these studies focusing on recruiting service users, the barriers identified could be equally applicable in recruiting family members.

Moreover, several studies have identified problems which are particularly prevalent in engaging the family members of FEP service users in research. Such challenges include: a lack of understanding of clinical research and potentially service provision;^13^, ^24^ diagnostic and prognostic ambiguity of FEP affecting family members' perception of their own needs of service;^11^, ^25^ previous experiences and degree of satisfaction with services generally, or aspects of care specifically;^13^ and also, more pressing demands, for example, needing to manage and cope with social and economic constraints or illness in other family members. While these challenges have mainly been investigated in the context of parent carers of FEP service users,^10^, ^11^, ^13^ it seems pertinent to extrapolate these findings to siblings, given that they may be attempting to attain independence from the family network, yet still heavily involved in supporting their sibling and family.^1^, ^3^, ^5^

Strengths of this study include that the process evaluation coincided with the active recruitment to the RCT, thus enabling participants to reflect on their practice and the current recruitment processes employed. Also, we actively sought to recruit staff from different disciplines, including PIs, clinicians and CSOs/CSAs, and from broad‐ranging recruitment sites, which facilitated an insight and multiple perspectives about this topic. However, we also acknowledge several study limitations, including the relatively small sample size and the lack of representation of staff from all recruitment sites. Despite data saturation being reached, it is possible that staff working at other sites may have had additional opinions and experiences different from results reported in here were not included. Exploring the views of siblings who participated in or declined to join the E Sibling RCT could have augmented the study findings, although this was not the remit of this study.

### 4.1 Clinical and research implications

Several strategies were identified which can enhance reach and recruitment of siblings to research. Arguably, these strategies are equally applicable in terms of boosting family inclusiveness in routine clinical settings. We suggest that undertaking a comprehensive family assessment, such as via the use of a genogram so as to map out familial networks and relationships, would be useful when the service user is first referred to EIPS.^34^, ^35^ Obtaining contact details for relevant family members including siblings seems warranted, consent‐permitting, partly so that they can be provided with information about services or means of accessing support. It may be that siblings (and family members in general) would benefit from health promotion activities, even offered via primary or voluntary sector where they are regarded as consumers in their own right.^35^ In terms of research, it is likely that specific family training with reference to good practice guidance in involving family members in research could be useful.^21^, ^36^ Such training can raise awareness of family members' needs and significance among both clinical and research staff, and to develop strategies to identify and approach family members more effectively. Joint‐working between R&D, CRN, clinicians and family support champions in such training would be particularly helpful in bridging the clinical and research activities. These different perspectives could help resolve the disparity in the interpretation of data governance policies and the communication and collaboration between clinical and research staff. Additionally, siblings should be involved in the design and dissemination of study methods, through patient and public involvement (PPI) activities, to help ensure that the study aims and procedures are developed effectively. Similarly, PPI endeavours could help to optimize that study information is communicated in a manner that takes into account the competing demands siblings have, and to enhance the reach and recruitment of studies.^19^, ^22^ Finally, issues pertaining to data protection and governance require consideration, so as to ensure that service users' rights are upheld, but also that family members' needs can be adequately assessed and met.^35^, ^36^ Future studies should bear these considerations in mind when planning and setting up the recruitment activities.

## Conflict of Interest

All authors declared no conflict of interest.

## Author Contributions

JS designed and undertook the study. JS and DS wrote the paper; CH and IN supervised the design and conduct of the research, edited and revised the paper; CG helped pilot the data collection procedures, check the data analysis and construct Table 2.

## References

[1] Smith J , Fadden G , O'Shea M . Interventions with siblings In: Lobban F, Barrowclough C, eds. A Casebook of Family Interventions for Psychosis. Chichester: Wiley and Sons; 2009:185–200.

[2] Sin J , Moone N , Harris P . Siblings of individuals with first episode psychosis—understanding their experiences and needs. J Psychosoc Nurs Ment Health Serv. 2008;46:33–40. 10.3928/02793695-20080601-11 18595457

[3] Bowman S , Alvarez‐Jimenez M , Wade D , Howie L , McGorry P . The impact of first episode psychosis on sibling quality of life. Early Interv Psychiatry. 2014;8:269–275. 24448630 10.1007/s00127-013-0817-5

[4] Smith MJ , Greenberg JS , Mailick Seltzer M . Siblings of adults with schizophrenia: expectations about future caregiving roles. Am J Orthopsychiatry. 2007;77:29–37. 17352582 10.1037/0002-9432.77.1.29 PMC2396553

[5] Sin J , Moone N , Harris P , Scully E , Wellman N . Understanding the experiences and service needs of siblings of individuals with first episode psychosis: a phenomenological study. Early Intervention in Psychiatry. 2012;6:53–59. 21952020 10.1111/j.1751-7893.2011.00300.x

[6] Sin J , Henderson C , Pinfold V , Norman I . The E Sibling Project—Development and exploratory randomised controlled trial of an online multi‐component psychoeducational intervention for siblings of individuals with first episode psychosis. BMC Psychiatry. 2013;13:123. 23622123 10.1186/1471-244X-13-123 PMC3644258

[7] Sin J , Henderson C , Norman I . Usability of online psychoeducation for siblings of people with psychosis. Int J Technol Assess Health Care. 2014;30:374–380. 25394550 10.1017/S0266462314000488

[8] Department of Health . Mental Health Policy Implementation Guide. London: DoH; 2001.

[9] National Institute for Clinical Excellence (NICE) . Psychosis and Schizophrenia in Adults—Treatment and Management (Clinical Guideline 176). London: NICE; 2014.

[10] Leavey G , Gulamhussein S , Papadopoulos C , Johnson‐Sabine E , Blizard B , King M . A randomized controlled trial of a brief intervention for families of patients with a first episode of psychosis. Psychol Med. 2004;34:423–431. 15259827 10.1017/s0033291703001594

[11] Kuipers E , Onwumere J , Bebbington P . Cognitive model of caregiving in psychosis. Br J Psychiatry. 2010;196:259–265. 20357299 10.1192/bjp.bp.109.070466

[12] Henderson C , Thornicroft G . Stigma and discrimination in mental illness: time to change. Lancet. 2009;373:1928–1930. 19501729 10.1016/S0140-6736(09)61046-1

[13] Szmukler G , Kuipers E , Joyce J , et al. An exploratory randomised controlled trial of a support programme for carers of patients with a psychosis. Soc Psychiatry Psychiatr Epidemiol. 2003;38:411–418. 12910336 10.1007/s00127-003-0652-1

[14] Howard L , de Salis I , Tomlin Z , Thornicroft G , Donovan J . Why is recruitment to trials difficult? An investigation into recruitment difficulties in an RCT of supported employment in patients with severe mental illness. Contemp Clin Trial. 2009;30:40–46. 10.1016/j.cct.2008.07.007 PMC2626649 18718555

[15] Woodall A , Morgan C , Sloan C , Howard L . Barriers to participation in mental health research: are there specific gender, ethnicity and age related barriers? BMC Psychiatry. 2010;10:103–112. 21126334 10.1186/1471-244X-10-103 PMC3016310

[16] Richards DA , Ross S , Robens S , Borglin G . The DiReCT study ‐ improving recruiting into clinical trials: a mixed methods study investigating the ethical acceptability, feasibility and recruitment yield of the cohort multiple randomised controlled trials design. Trials. 2014;15:398. 25318374 10.1186/1745-6215-15-398 PMC4210622

[17] Steckler A, Linnan L, eds. Process Evaluation for Public Health Interventions and Research. San Francisco: Jossey‐Bass; 2002.

[18] Callaghan P , David A , Lewis S , et al. Developing support for mental health clinical research: the Mental Health Research Network experience. J Clin Investig. 2012;2:459–463.

[19] Ennis L , Wykes T . Impact of patient involvement in mental health research: longitudinal study. Br J Psychiatry. 2013;203:1–6. 24029538 10.1192/bjp.bp.112.119818

[20] Staley K . A Series of Case Studies Illustrating the Impact of Service User and Carer Involvement on Research. London: National Institute for Health Research (The Mental Health Research Network); 2013.

[21] Kara H . CRN: West Midlands Mental Health Carer Reference Group. Birmingham: NIHR CRN: MH; 2014 http://www.crn.nihr.ac.uk/wp-content/uploads/mentalhealth/CRN%20West%20Midlands%20Carer%20Reference%20Group%20Evaluation%20-%20Final%20Report%20PDF.

[22] Ritchie J , Lewis J , O'Connor W . Carrying out qualitative analysis In: Ritchie J, Lewis J, eds. Qualitative Research Practice: A Guide for Social Science Students and Researchers. London: SAGE Publications Ltd; 2003:219–262.

[23] Green J , Thorogood N . Qualitative Methods for Health Research. London: SAGE Publications; 2004.

[24] Oakley A , Strange V , Bonell C , Allen E , Stephenson J , RIPPLE Study Team . Process evaluation in randomised controlled trials of complex interventions. Br Med J. 2006;332:413–416. 16484270 10.1136/bmj.332.7538.413 PMC1370978

[25] Onwumere J , Kuipers E , Bebbington P , et al. Caregiving and illness beliefs in the course of psychotic illness. Can J Psychiatry. 2008;53:460–468. 18674404 10.1177/070674370805300711

[26] Department of Health . Research Funding and Priorities. London: Department of Health; 2010.

[27] Department of Health . No Health Without Mental Health—A Cross‐Government Mental Health Outcomes Strategy for People of All Ages. London: DOH; 2011.

[28] Patterson S , Kramo K , Soteriou T , Crawford MJ . The great divide: a qualitative investigation of factors influencing researcher access to potential randomised controlled trial participants in mental health settings. J Ment Health. 2010;19:532–541. 21121823 10.3109/09638237.2010.520367

[29] McDonald A , Knight R , Campbell M , et al. What influences recruitment to randomised controlled trials? A review of trials funded by two UK funding agencies. Trials. 2006;7:9. 16603070 10.1186/1745-6215-7-9 PMC1475627

[30] Borschmann R , Patterson S , Poovendran D , Wilson D , Weaver T . Influences on recruitment to randomised controlled trials in mental health settings in England: a national cross‐sectional survey of researchers working for the Mental Health Research Network. BMC Med Res Methodol. 2014;14:23. 24533721 10.1186/1471-2288-14-23 PMC3928923

[31] Fletcher B , Gheorghe A , Moore D , Wilson S , Damery S . Improving the recruitment activity of clinicians in randomised controlled trials: a systematic review. BMJ Open. 2012;2:e000496. 10.1136/bmjopen-2011-000496 PMC3253423 22228729

[32] Mitchell A , Gill J . Research productivity of staff in NHS mental health trusts: comparison using the Leiden method. Psychiatr Bull. 2014;38:19–23. 10.1192/pb.bp.113.042630 PMC4067840 25237485

[33] Docherty M , Thornicroft G . Specialist mental health services in England in 2014: overview of funding, access and level of care. Int J Ment Health Syst. 2015;9:34. 26322123 10.1186/s13033-015-0023-9 PMC4553216

[34] Sin J , Spain D , Jordan C , Griffiths C . Siblings of people with severe mental illness. J Ment Health Train Educ Pract. 2014;9:215–221.

[35] Sin J , Murrells T , Spain D , Norman I , Henderson C . Wellbeing, mental health knowledge and caregiving experiences of siblings of people with psychosis, compared to their peers and parents: an exploratory study. Soc Psychiatry Psychiatr Epidemiol. 2016;51:1247–1255. 27121259 10.1007/s00127-016-1222-7 PMC5025483

[36] Mental Health Research Network (MHRN) . Good Practice Guidance for Involving Carers, Family Members and Close Friends of Service Users in Research. London: National Institute for Health Research, MHRN; 2012.

